# Kilogram‐Scale Crystallogenesis of Halide Perovskites for Gamma‐Rays Dose Rate Measurements

**DOI:** 10.1002/advs.202001882

**Published:** 2020-12-09

**Authors:** Pavao Andričević, Pavel Frajtag, Vincent Pierre Lamirand, Andreas Pautz, Márton Kollár, Bálint Náfrádi, Andrzej Sienkiewicz, Tonko Garma, László Forró, Endre Horváth

**Affiliations:** ^1^ Laboratory of Physics of Complex Matter (LPMC) Ecole Polytechnique Fédérale de Lausanne Centre Est, Station 3 Lausanne CH‐1015 Switzerland; ^2^ Laboratory of Reactor Physics and Systems Behaviour Ecole Polytechnique Fédérale de Lausanne Centre Est, Station 3 Lausanne CH‐1015 Switzerland; ^3^ ADSresonances Sàrl Route de Genève 60B, CH‐1028 Préverenges Switzerland; ^4^ Power Engineering Department Faculty of Electrical Engineering Mechanical Engineering and Naval Architecture University of Split Ulica Ruđera Boškovića 32 Split 21000 Croatia

**Keywords:** dosimetry, operational stability, perovskite gamma detection, record crystal size, self‐healing

## Abstract

Gamma‐rays (*γ*‐rays), wherever present, e.g., in medicine, nuclear environment, or homeland security, due to their strong impact on biological matter, should be closely monitored. There is a need for simple, sensitive *γ*‐ray detectors at affordable prices. Here, it is shown that *γ*‐ray detectors based on crystals of methylammonium lead tribromide (MAPbBr_3_) ideally meet these requirements. Specifically, the *γ*‐rays incident on a MAPbBr_3_ crystal generates photocarriers with a high mobility‐lifetime product, allowing radiation detection by photocurrent measurements at room temperatures. Moreover, the MAPbBr_3_ crystal‐based detectors, equipped with improved carbon electrodes, can operate at low bias (≈1.0 V), hence being suitable for applications in energy‐sparse environments, including space. The *γ*‐ray detectors reported herein are exposed to radiation from a ^60^Co source at dose rates up to 2.3 Gy h^−1^ under ambient conditions for over 100 h, without any sign of degradation. The excellent radiation tolerance stems from the intrinsic structural plasticity of the organic–inorganic halide perovskites, which can be attributed to a defect‐healing process by fast ion migration at the nanoscale level. The sensitivity of the *γ*‐ray detection upon volume is tested for MAPbBr_3_ crystals reaching up to 1000 cm^3^ (3.3 kg in weight) grown by a unique crystal growth technique.

## Graphical abstract

The largest halide perovskite crystal specimens are produced up to 3.3 kg.  The increased size allows fabrication of sensitive *γ*‐ray detectors able to work under high dose rates (2.3 Gy h^−1^) for over 100 h, without any sign of degradation. The excellent radiation tolerance can be attributed to a defect‐healing process by fast ion migration at the nanoscale level.

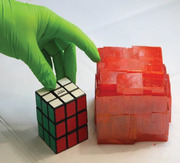

Currently, radiation detectors are extensively used in various domains, including industry, nuclear power plants, homeland security and defense, environmental monitoring, academic research, and healthcare as well, e.g., cancer development and osteoporosis detection and monitoring. For efficient high‐energy‐photon harvesting, conventionally used semiconductor materials must have multiple distinct characteristics, such as large detection volume to intercept radiation, an elevated linear attenuation coefficient, large and balanced carrier mobilities (***μ***) concomitant with long charge carrier lifetimes (***τ***), thus leading to high values of their product (***μτ***), which is a prerequisit for efficient charge collection. This requires high crystal quality, a low charge trap density, and as little as possible of impurities, concomitant with a high resistivity.^[^ ^1^, ^2^ ^]^ Nowadays, the highest values of the ***μτ*** product are obtained in conventional crystalline semiconductor materials (commercialized already in the 1970s), in which carrier transport is not limited by scattering and trapping at grain boundaries.^[^ ^3^ ^]^ However, the availability of such semiconductors, especially with high atomic numbers (high‐***Z***), in sufficiently large, single‐crystalline forms, which would also be both chemically and mechanically robust, is still limited.^[^ ^4^ ^]^ High‐purity large germanium single crystals (Ge SCs) possess the best electronic properties for harvesting of high‐energy‐photons.^[^ ^5^ ^]^ However, the operation of Ge SCs‐based detectors is possible only at or below the liquid nitrogen temperature due to the narrow bandgap of germanium.

Among non‐cooled semiconductor radiation detectors most noteworthy are CdTe, HgI_2_, GaAs, and the frontrunner zinc‐alloyed CdTe (Cd_1−_ *_x_*Zn*_x_*Te, denoted CZT for 0 < *x* < 0.2). CZT single crystals (SCs) exhibit the highest resolution of *γ*‐ray spectra due to their wide band‐gap of above 1.6 eV, a high resistivity up to (10^8^–10^9^) Ω cm at room temperature and a large ***μτ*** product.^[^ ^1^ ^]^ Nevertheless, some important limitations of CZT detectors are related to a relatively low hole mobility, a cost‐restricted crystal manufacturing at a scaled‐up level and a high operational voltage (50 V and up).

The recently rediscovered metal halide perovskites (MHPs) have been found to meet key requirements for high‐energy radiation detection.^[^ ^1^, ^2^, ^4^, ^6^, ^7^, ^8^, ^9^, ^10^, ^11^, ^12^, ^13^, ^14^ ^]^ In particular, the high‐***Z*** chemical elements, such as lead (Pb), iodine (I), and bromine (Br), concomitant with relatively large densities of MHPs (≈4.0 g cm^−3^), allow for a substantial attenuation of high‐energy photons (e.g., for Cs_0.1_FA_0.9_PbI_2.8_Br_0.2_ SCs the ***μτ*** product is of ≈ 1.2 × 10^−1^ cm^2^ V^−1^).^[^ ^9^ ^]^ Unlike the market‐leading crystals, these MHP SCs may grow from abundant and low‐cost raw materials in solutions at near room temperature without using high capital demanding infrastructures.^[^ ^1^ ^]^ Exposed to X‐rays,^[^ ^15^, ^16^, ^17^, ^18^, ^19^, ^20^, ^21^ ^]^ detection of dose rates lower than the typically required in medical diagnostics (≈5.5 μGy_air_ s^−1^) has been demonstrated.^[^ ^22^ ^]^ Exposed to a ^137^Cs source *γ*‐ray radiation, the first MAPbI_3_ SC photodetectors have shown a photon‐to‐electron conversion efficiency of 3.9%^[^ ^6^ ^]^ and laboratory prototyped MHP‐based *γ*‐ray counting dosimeters have exhibited excellent sensitivity, high resistivity (3.6 GΩ cm) and high values of the ***μτ*** product (1.8 × 10^−2^ cm^2^ V^−1^,^[^ ^1^, ^7^ ^]^) enabling a remarkable energy resolution.^[^ ^10^, ^23^, ^24^ ^]^ Because of the high intrinsic resistivity of MHPs, the operation of MHPs‐based radiation detectors requires bias voltages above 5 V. Consequently, ion migration and detrapping of deep charge traps may occur, thereby increasing the dark current, noise and induce hysteretic behavior.^[^ ^25^ ^]^ To date, the volume of the laboratory‐grown MHP SCs dedicated for *γ*‐ray detecting purposes does not exceed 1.2 cm^3^. Therefore, advancements in low‐temperature solution‐grown crystal growth of large MHP SCs may be a major stepping‐stone to stimulate further research and reduce the technological barriers for a successful implementation of MHPs in high‐energy radiation detection.

In this study, we introduce the “oriented crystal‐crystal intergrowth” method, hereinafter referred to as oriented crystal‐crystal intergrowth (OC2G) technique, which yielded solution‐grown MAPbBr_3_ crystals with volume and mass of over 1000 cm^3^ and 3 kg, respectively. Using a broad range of MAPbBr_3_ SCs contacted with various carbon electrodes, we demonstrate operationally stable *γ*‐rays detection from a ^60^Co source under ambient conditions, with dose rates in the 0.07–2.30 Gy h^−^^1^ range. The measured dose rates show a good correlation with commercially available thermoluminescent dosimeters (Harshaw TLDs‐700, 4.38% ^7^Li‐doped) and a calibrated *γ*‐sonde (Berthold LB 6414). Furthermore, the devices exhibit operational stability for over 100 h. The unexpectedly high tolerance of MAPbBr_3_ crystals to *γ*‐radiation can be attributed to their intrinsic compositional plasticity, namely a thermodynamically favorable chemical process of “radiation‐damage‐phase‐separated” components (MABr and PbBr_2_) to perovskite product on a nanoscale level.

The growth of MAPbBr_3_ SCs was performed using the inverse temperature crystallization method.^[^ ^26^, ^27^ ^]^ First, small MAPbBr_3_ SCs with sizes of ≈5 mm were grown by gradually increasing the temperature of the room temperature saturated MAPbBr_3_ solution using an oil bath. Subsequently, these crystals were used as seed crystals to grow even larger MAPbBr_3_ single crystals via a “suspended seed crystal” technique, as depicted in **Figure** **1**a. In OC2G, these building blocks were aligned side‐by‐side along with their facets, just as a Rubik's Cube pattern, and fused together by the inverse temperature crystallization. The diagrams and photographic images explaining the consecutive stages of growing large MAPbBr_3_ crystals using the OC2G technique are shown in Figure 1b,c. The corresponding time‐lapse video clip can be found in the Supporting Information. Briefly, the herein described OC2G technique yields large MAPbBr_3_ crystals, weighing over 3 kg and having the edge lengths of over 10 cm (Figure 1d). To the best of our knowledge, these are the largest MHP crystals produced so far. It is also worth mentioning that, in principle, there are no technological limitations to further exceed these impressive crystal sizes in future.

**Figure 1:**
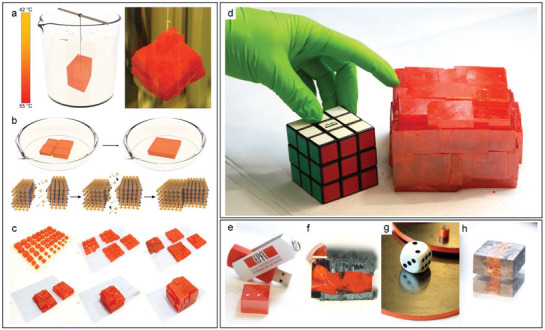
The “oriented crystal‐crystal growth” (OC2G) method of large MAPbBr_3_ crystals and strategies for making electrical contacts. a) Growing of large crystals by the suspended seed crystal technique; b,c) Diagrams and photographic images of the consecutive steps of fusing together individual single crystals into a large crystal. d) The final result: a 3.3 kg weighting crystal of MAPbBr_3_; e–h) Illustration of sample contacting strategies: (e) silver paste contacts, (f) aligned carbon nanotube contacts engulfed by the crystal, (g) pressed carbon paper on the surface of the compressed polycrystalline sample, and (h) graphite spray contacts.Rubik’s Cube is a registered trademark and used by permission of Rubik’s Brand Ltd, www.rubiks.com.

For direct *γ*‐radiation detection, the elaboration of stable and low resistive contacts is very important. Our detector devices were initially assembled by using gold wires as electrical contacts, which were attached to the surface of MAPbBr_3_ SCs with conductive adhesives, like, e.g., Dupont 4929 silver epoxy (Figure 1e). However, it has been recently recognized that perovskite‐based optoelectronic devices implementing silver electrodes exhibited stability issues in long‐term operation. Notably, silver and other noble metal‐based electrodes undergo electrocorrosion, thus degrading and weakening the electronic properties.^[^ ^28^ ^]^ Therefore, lately, carbon‐derived components such as carbon nanotubes, graphene, reduced graphene oxide, fullerenes, and graphite have been proposed.^[^ ^29^, ^30^, ^31^, ^32^ ^]^ Here, three types of carbon electrodes were investigated. The first type was the vertically aligned carbon nanotube (VACNT) forest, where the perovskite single crystal has engulfed the individual nanotubes as protogenetic inclusions (Figure 1f), thus leading to the formation of a three‐dimensionally enlarged interface.^[^ ^29^ ^]^ The second kind of electrodes was a graphite paper in pressed MAPbBr_3_ polycrystalline sample (Figure 1g). The third type was the graphite spray covering the whole surface of the large MAPbBr_3_ single crystals allowing large electrode surfaces (Figure 1h). As demonstrated in the following section, the graphite spray contacts turned out as the optimal solution.

The *γ*‐rays detection was studied by exposing the fabricated devices to radiation from a 269 GBq ^60^Co source (1.25 MeV) inside an irradiation cavity “LOTUS” (Laboratory for Reactor Physics and System Behaviour at the EPFL), as shown for a device with graphite spray electrodes in **Figure** **2**a. Samples were positioned in a distance of 25–125 cm from the source, thus exposing them to the corresponding dose rates from 0.07 to 2.30 Gy h^−1^. It is worth noting that the allowed annual occupational dose limit of gamma radiation (total effective dose equivalent) is of 50 mSv; Gy = Sv (if absorbed by the body).^[^ ^33^ ^]^

**Figure 2:**
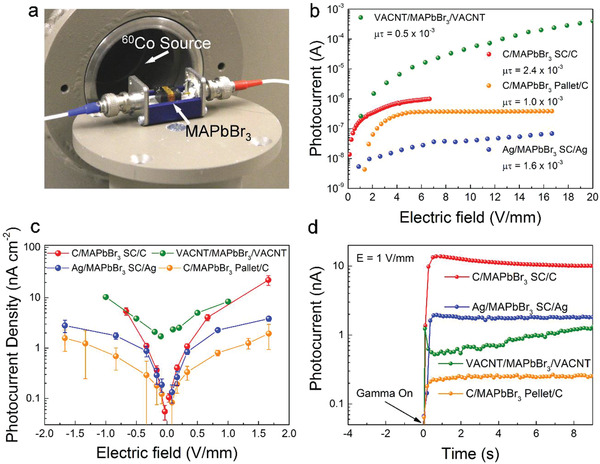
Comparison of performances of MAPbBr_3_ *γ*‐ray photodetectors with various kinds of contact electrodes. a) Image of the detector device inserted in the cavity and exposed to a ^60^Co *γ*‐source. b) Photocurrent versus voltage measured at room temperature for the extraction of mobility‐lifetime for holes according to Equation 1. c) The electric field dependence of photocurrent under a 2.3 Gy h^−1^ dose rate for the four device architectures, showing an excellent resolution even at low electric fields. d) Time dependence of the photocurrent responses at 1 V mm^−1^ electric field. For simplicity we will use: gamma on – source out, direct exposure; gamma off – source shielded.

To compare the different devices, the fundamental figure of merit for *γ*‐radiation detectors, i.e., the ***μτ*** product, was calculated from the *γ*‐photocurrent–voltage curves shown in Figure 2b. The ***μτ*** product provides an estimate of the ability of charge carriers in a crystal of a given size to reach the current collectors before recombination.^[^ ^1^ ^]^ The obtained ***μτ*** products range from 0.5 × 10^−3^ to 0.3 × 10^−2^ cm^2^ V^−1^, which are comparable to the highest quality CZT SCs of 0.91 × 10^−2^ cm^2^ V^−1^.^[^ ^34^ ^]^ However, the actual advantage of MAPbBr_3_ over CZT is that it possess comparable ***μτ*** products for both holes and electrons,^[^ ^12^ ^]^ even though MAPbBr_3_ has shown to be a hole transport semiconductor with electrons mostly affected by traps.^[^ ^23^, ^35^ ^]^ CZT, on the other hand, has one order of magnitude lower hole mobility.^[^ ^36^ ^]^ Furthermore, these values are in the range with ***μτ*** products of other hybrid perovskite detectors, keeping in mind they were extracted from photocurrents generated from high‐energy photons compared to previous reports that use visible light or X‐ray irradiation. Additionally, unlike CZT, which requires fairly high bias voltages (50–1000 V), the C/MAPbBr_3_ SC devices show a high sensitivity even at low electric fields of 0.1 V mm^−1^. This low bias voltage (0.1–5 V) offers the possibility of application in energy‐sparse environments, for example in space.^[^ ^37^ ^]^ Furthermore, its maximum photocurrent response of 2.3 µA (Table S1, Supporting Information) reached in less than 500 ms (Figure 2d) is in range with the best values acquired for MHP‐based gamma detectors.

More details showing the responses of the various devices to *γ*‐rays radiation can be found in Figures S1–S4 and Table S2 in the Supporting Information. As seen in Figure 2c, the two best‐performing devices are fabricated with the VACNT and graphite spray electrodes. The VACNT/MAPbBr_3_ SC/VACNT device configuration (Figures S2 and S3, Supporting Information) offers the highest photocurrent density at low electric fields, presumably due to the large contact area between the CNTs and the perovskite, as well as the field enhancement factor at the nanotube tips.^[^ ^32^ ^]^ However, due to the complexity of the device fabrication larger devices with VACNT electrodes are hard to obtain. Therefore, the absolute value of photocurrent response is much higher in devices fabricated with the graphite spray electrodes (Figure 2d), because of their larger size allowing more interactions of high‐energy photons with the device active area. In addition, the VACNT electrode device has unstable currents in time as seen in Figure 2d. These VACNTs have shown to enhance ion migration in perovskite single crystals.^[^ ^32^ ^]^ This property utilized to an advantage for light emission is here, unfortunately, causing an unwanted current drift in detectors and should be reduced as much as possible. One can notice that even the “low budget” device consisting of the pressed and sintered polycrystalline perovskite with graphite paper might perform properly (Figure S4, Supporting Information). Therefore, most of the results hereafter are shown for the best performing devices, i.e., based on MAPbBr_3_ SCs contacted with graphite electrodes. Still, to determine the geometrical dependence of the detection (surface versus volume) silver contacts were used as well due to easy contact reproducibility.

Perovskite devices of the same architecture but with different sizes of MAPbBr_3_ SCs were placed in the irradiation cavity to study phenomena related to the size and orientation of crystals to the incident radiation. Initially, a photodetector device with silver electrodes was exposed to both *γ*‐rays and visible light illumination at different crystal orientations, thus allowing for exposure of different crystal surfaces, as shown in the diagrams shown in Figure S5a in the Supporting Information. As expected, under visible light illumination, the photocurrent markedly increased when a larger active surface of the MAPbBr_3_ SC was exposed. In contrast, the photocurrent response to *γ*‐irradiation exhibited a negligible difference between the two orientations (Figure S5b, Supporting Information). Additionally, detectors designed around larger MAPbBr_3_ SCs, having the same spacing between the silver electrodes, were exposed to the *γ*‐source, as schematically shown in Figure S5c in the Supporting Information. This approach allowed us to measure responsivity of *γ*‐ray detectors as a function of the volume of MAPbBr_3_ SCs in range of 141–16 800 mm^3^.

As shown in Figure S5d (Supporting Information), the photocurrent is markedly growing with the increasing crystal volumes of MAPbBr_3_ SCs. Initially, for MAPbBr_3_ SCs of smaller sizes (up to ≈ 2000 mm^3^), the photocurrent increases linearly with growing crystal volumes, thus confirming both a high photocarrier extraction efficiency and the sensitivity dependence on the crystal volume. As might be expected, however, due to the finite value of the ***μτ*** product, as well as simplicity of our detector (employing just two electrodes placed on one crystal facet) this dependence becomes sublinear with further increasing the crystal volumes.

To estimate what thickness of SCs is needed for efficient interaction of high‐energy photons with the active material the penetration depth was calculated. For the Co‐60 gamma source, a linear attenuation coefficient of 0.1917 cm^−1^ was estimated which corresponds to a penetration depth of 5.2165 cm. In Figure S6 (Supporting Information) this value was compared to those of other common radiation detection materials. This proves that multiple‐centimeter crystal sizes would be needed for the detection of photons with above 1 MeV energies.

Therefore, to examine the limitations of the volume dependence, the OC2 growth has been developed, yielding the largest perovskite crystals thus far. The 3.8 kg crystal with a volume of over one cubic decimeter and a thickness over 10 cm, shown in Figure 1d, was tested for gamma detection. First, silver epoxy contacts were deposited, as in our previous samples (Figure S7, Supporting Information). However, it is obvious that a more volumetric electrode design is needed to be able to collect all generated charges. Therefore, the focus was moved to the graphite spray electrode architecture. Photocurrents of over 100 nA were acquired at relatively low bias voltages, 2–5 V, as seen in Figure S8 in the Supporting Information. These values were compared to other graphite spray electrode samples based on MAPbBr_3_ SCs, grown by inverse temperature crystallization. As seen in **Figure** **3**, an increase in the photoresponse of over 100 times is present when utilizing crystals grown by the OC2G method. Even despite a lower dose‐rate of irradiation due to setup limitations. Therefore, once again the importance of an increased volume is confirmed, as well as the fact that the gain is not linear. Clearly, a loss of photocarriers, which cannot be harvested by the electrodes due to recombination or trapping within the active crystal volume, is present. This problem can be solved by designing a truly volumetric charge collection pattern. Nevertheless, the OC2G crystal, even with this simple electrode design, exhibited a significant signal increase and is proving to be a viable method for future large volume perovskite radiation detectors. Additionally, due to the high density of perovskite, large‐volume MAPbBr_3_ crystals could offer other applications, such as radiation shielding (Figure S9, Supporting Information). For example, the 12 cm thick MAPbBr_3_ crystal can attenuate 93.5% of the 269 GBq ^60^Co (1.25 MeV) source. In addition, there is no technological limitation to further increase this size.

**Figure 3:**
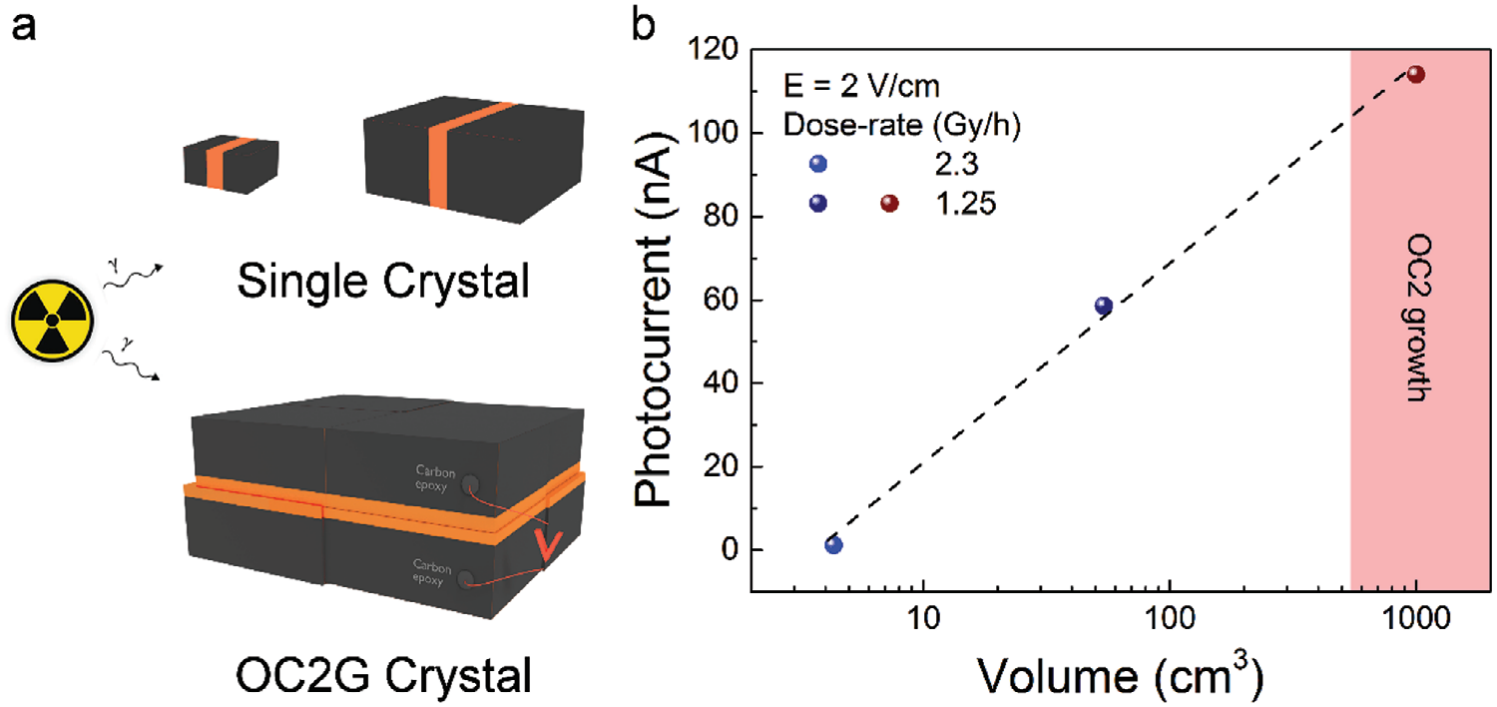
The size effect of perovskite‐based gamma detectors. a) Schematic illustration of the configuration of volume dependence measurements of the photocurrent of MAPbBr_3_ detectors based on single crystals attained by the inverse temperature crystallization method and crystals attained by the OC2G method. b) Photocurrent dependence as a function of the crystal volume.

It is well known that MHPs are prone to ionic migration under an applied electric field which alters the electronic response of these materials. This is visible in **Figure** **4**a which shows the photocurrent transient responses with on‐off radiation cycles under an applied field of 0.5 V during *γ*‐rays exposure. We must emphasize once again, the nice feature that the MAPbBr_3_ SC‐based detector can work for both bias voltage polarities. After the initial rapid rise of the photocurrent, it starts to slowly decay in time, together with the change of the dark current (with *γ*‐source shielded). This is the consequence of the ion migration under the applied field. As shown in Figure 4b and Figure S11 (Supporting Information), this effect is present in all applied fields to some extent. The device was operated at 0.1–5 V bias voltages, yielding photocurrents from 0.43 to 88.15 nA (Figure 4b). These photocurrent values are reached in less than one second, as is in the case of visible light detection. Although, there is still a drift in the current, at the rising edge the photocurrent still allows precise radiation detection, which could be improved even further if the time during which the sampling voltage is applied is reduced to, e.g., couple of seconds or less.

**Figure 4:**
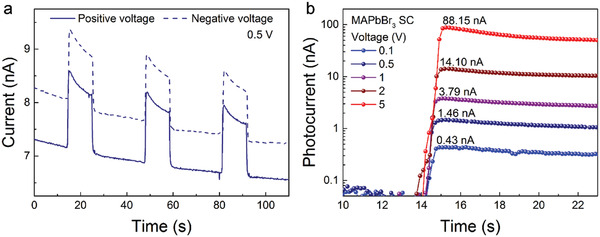
Performance of the *γ*‐rays photodetector. a) Time dependence of the current under a 2.3 Gy h^−1^ dose rate for 0.5 V bias voltage of both polarities. The signal and the base‐line (dark current) shift are due to ion migration under the applied field b) Zoom on the leading edge of the signal at various bias voltages for 2.3 Gy h^−1^ dose rate.

Dose rate‐dependent measurements, usually neglected in most of the reports, are important for the characterization of the device performance. In the case of a fixed activity *γ*‐source, the dose could be changed by the distance between the device and the irradiation source, this way varying the number of *γ*‐photons reaching the detector in a unit time. Hence, our detector was positioned at different distances from the source, ranging from 25 to 125 cm. Corresponding values of dose rates are ranging from 2.30 to 0.07 Gy h^−1^, which were measured by a calibrated *γ*‐sonde. On–off characteristics are shown in **Figure** **5**a (with multiple measurements shown in the inset) for multiple distances from the source under a bias voltage of 1 V, at which the “dark” current is predominantly stable.

**Figure 5:**
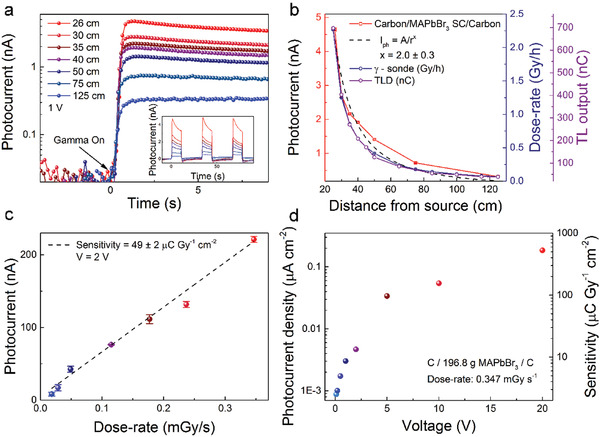
Dose rate measurements. a) Time dependence of the photocurrent responses for different distances from the source. The inset shows a sequence of the photocurrent transients measured while turning gamma radiation ON and OFF for the same set of source‐detector distances. b) Comparison of the detector photocurrent with commercially available and calibrated detectors with a fit to the $\dot{D}$_0_ */r^x^* function. c) Detector sensitivity determined from the photocurrent dose‐rate dependence and d) its change with voltage for the *γ*‐ray photodetector device with graphite spray electrodes.

The value of the photocurrent at the leading edge of the *γ*‐ray on was plotted as a function of distance from the source (Figure 5b) and compared to two types of commercially available detectors for gamma dose rate measurements: thermoluminescent dosimeters (Harshaw TLDs‐700, 4.38% ^7^Li‐doped) and a calibrated *γ*‐sonde (Berthold LB 6414). All the measured values show a strong correspondence. Furthermore, the results are in a good agreement with the theoretical inverse‐square law behavior (black dashed line). Fitted to the$\dot{D}$_0_ */r^x^* function, where $\dot{D}$_0_is the dose rate at the distance *r* and *x* is the power‐constant. The extracted value of the exponent *x* is (2.0 ± 0.3). However, the irradiator with the ^60^Co radioactive source is placed in a concrete irradiation cavity of reduced dimensions (3.6 × 2.4 × 3 m^3^), and therefore, the shielding geometry has to be taken into account. An intense scattering of gamma particles from the concrete walls affects the theoretically estimated inverse‐square law behavior of dose rates for a point‐like source, in open space.

From the photocurrent responses at different dose‐rates acquired by the calibrated detectors, one can calculate the sensitivity of detecting gamma irradiation. The sensitivity was estimated from the slope of the linear fit of the photocurrent dose‐rate dependence. Values of 112 ± 3 and 49 ± 0.2 µC Gy^−1^ cm^−2^ were determined, for our two best samples, MAPbBr_3_ SC with VACNTs (Figure S12a, Supporting Information) and MAPbBr_3_ SC with graphite spray electrodes (Figure 5c; Figure S13a, Supporting Information), respectively. It is important to point out how these sensitivities are achieved at low bias voltages (<2 V) and can be enhanced by increasing the bias voltage. As seen in Figure 5d, sensitivity values as high as 531 µC Gy^−1^ cm^−2^ can be attained for the MAPbBr_3_ SC gamma detector with graphite spray electrodes at 20 V (334 µC Gy^−1^ cm^−2^ for VACNTs at 0.5 V, Figure S12b, Supporting Information). These values are one order of magnitude higher than the best reported so far sensitivity of perovskite detectors exposed to *γ*‐rays, of 41 µC Gy^−1^ cm^−2^.^[^ ^10^ ^]^ Moreover, they are reaching the sensitivity values of CZT‐based X‐ray detectors used for medical imaging.^[^ ^38^ ^]^

Radiation damage is one of the major concerns of the state‐of‐the‐art commercially available detectors. It is well known that neutrons, protons, electrons, and even *γ*‐radiation can lead to displacement damage in solid‐state detectors (Si, CZT, etc.).^[^ ^39^ ^]^ In the case of high‐energy particles, the displacement damage is a cascade‐type process involving multiple interactions, resulting in an extended damage region or defect clusters. In contrast, in the case of *γ*‐radiation, the displacement damage is caused by high‐energy Compton electrons (≈1 MeV) that only produce point defects.^[^ ^40^ ^]^ These displacements damages lead to an increase in the leakage current and damage the sensor. Conventional materials for *γ*‐rays detection can be made more radiation‐resistant by a purposeful introduction of known impurities.^[^ ^41^ ^]^ Conceivably, certain impurities, by their interaction with the primary defects caused by radiation, vacancies and interstitials, can neutralize them without significantly altering the material properties. For example, silicon detectors with enhanced oxygen content exhibited better radiation hardness, being almost insensitive to *γ*‐rays radiation up to 6 MGy^[^ ^39^ ^]^

As compared to the robust Si and CZT, MHPs are considered even more prone to degradation when exposed to various environmental factors. Consequently, performance deterioration of the MHP‐based *γ*‐rays detector can clearly be expected in long‐term operation. Therefore, since the detector stability against *γ*‐radiation is an important factor, we have performed test measurements on the time‐scale of 100 h of operation. Measurements were done for the MAPbBr_3_ SC device with graphite spray electrodes at the strongest dose rate of 2.3 Gy h^−1^ and under a bias voltage of 1 V applied during 1 s in every minute (hereinafter referred to as a pulsing voltage signal, Figure S14, Supporting Information). This way one can reduce the dark current variation due to the poling effect on ion migrations (**Figure** **6**a). After the initial decrease of photocurrent (commonly seen in perovskite‐based devices), it shows remarkable stability up to 100 h, the time scale of the measurement. The weak variation of the photocurrent in time, which is caused by ion migration inside the perovskite single crystal, can in principle be suppressed by using a low‐frequency pulsed voltage source to operate the detector.

**Figure 6:**
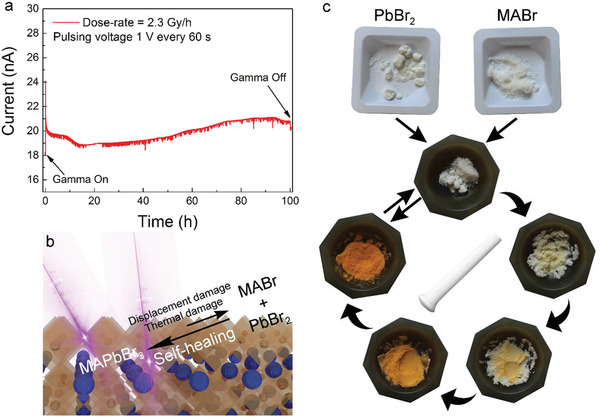
Long‐term operational stability. a) Photocurrent measurement under a pulsed 1 V bias voltage of a MAPbBr_3_ device exposed to 2.3 Gy h^−1^ dose rate for 100 h. b) Schematic representation of the self‐repairing behavior of the perovskite under gamma irradiation. c) Demonstration of a solvent‐free, solid‐state PbBr_2_ and MABr reaction and a formation of MAPbBr_3_ obtained by mechanical grinding, as a possible model of the fast self‐healing capability of the material after radiation damage.

The physical mechanism behind the good stability under irradiation in MHPs is currently unknown. It has been reported, that MHPs possess natural defects, which are known not to interfere with detection sensitivity. These defects may act in the same way as the aforementioned purposely introduced impurities in Si, likewise improving the radiation hardness of MHPs single crystals. Another more important feature is that, unlike the robust semiconductors Si and CZT, MHPs are “soft mixed ionic and electronic semiconductors.” When atoms are knocked out from their equilibrium positions, they form point defect like vacancies. This can, for example, create bromine rich and bromine deficient parts (detailed equation in the Supporting Information). Which can further lead to a complete separation, resulting in the production of the MAPbBr_3_ degradation compounds, MABr and PbBr_2_.^[^ ^42^ ^]^ Accompanied by a phase transitions and decreased absorption, which have already been observed in perovskites after long‐term irradiation,^[^ ^43^ ^]^ this would result in deterioration of the device performance. However, perovskites have shown the possibility to revert to their initial condition after or even during these long‐term operations, known as the self‐healing mechanism.^[^ ^44^, ^45^, ^46^ ^]^ Migration of the charged defects^[^ ^41^ ^]^ or their (trans)formation by chemical reactions^[^ ^44^ ^]^ are postulated to be the cause of this behavior (see the sketch of Figure 6b). As a proving example, experiments showed that perovskite solar cells survived accumulated dose levels up to 10^16^ and 10^15^ particles per cm^2^ of electrons (1 MeV) and protons (50 KeV), respectively, which are known to completely destroy crystalline Si‐, GaAS‐, and InGaP/GaAs‐based solar cells.^[^ ^47^ ^]^ Besides, perovskite solar cells have shown to retain 96.8% of their initial power conversion after more than 1500 h of continuous gamma irradiation, with and accumulated dose of 23 kGy.^[^ ^45^ ^]^ Yet, the mechanism behind this long‐term *γ*‐radiation stability under operational conditions and very high irradiation dose rates has not been fully addressed. The structural plasticity of organic–inorganic halide perovskite, fast ion migration and thermodynamically favorable chemical reaction of “radiation‐damage‐phase‐separated” components (MABr and PbBr_2_) to perovskite product stimulate this reversible self‐healing process on the nanoscale. To demonstrate the affinity of these components to enter into the reaction in solid‐state, without solvent, just by steric proximity promoted by mechanical mixing is shown in Figure 6c. One can see that after several minutes of grinding macroscopic quantities, the orange–yellow color of MAPbBr_3_ is obtained. Therefore, even if a complete degradation of the material would occur, in a confined space of the crystal (at a microscopic scale), this mechanism might induce the self‐healing of the radiation damage.

Shelf‐life stability is a further issue of MAPbBr_3_ crystal‐based *γ*‐ray detectors, due to their instability to environmental conditions. However, detectors protected against humidity and oxygen by encapsulation in polydimethylsiloxane (PDMS) remained operationally stable under the shelf‐life conditions for over 1 year (Figure S15, Supporting Information). Additionally, this polymer coating layer allows easier handling of the detector device, lowering the risk of lead toxicity.

Detectors based on MAPbBr_3_ SCs with carbon electrodes demonstrate an operational stability in *γ*‐radiation dose rate measurements in the emission range of 0.07–2.30 Gy h^−1^ from a ^60^Co radiation source under ambient and operational conditions for a testing period of 100 h. No degradation or deterioration of *γ*‐radiation detection capabilities is observed during the testing period. We attribute the observed excellent radiation tolerance to the intrinsic structural plasticity of MHPs, fast ion migration accompanying a reversible component phase separation based defect‐healing process on a nanoscale. Photocurrent responses of the devices to *γ*‐radiation are directly proportional to the active volume of MAPbBr_3_ SCs. For this reason, we have developed a crystal growth technique OC2G which was efficiency demonstrated by a crystal of 3.3 kg. Our detector was benchmarked to commercial devices. The simplicity, low‐cost solution‐based fabrication process and operational stability under *γ*‐irradiation of the crystalline MAPbBr_3_ make this material a good candidate for a new generation of high‐energy radiation detectors. Additionally, the above demonstration of kilogram scale crystallogenesis of halide perovskites coupled with future cutting and slicing technologies will enable the development of crystalline perovskite wafers for various optoelectronic application.

## Experimental Section

##### Growth of MAPbBr_3_ SC

The MAPbBr_3_ in dimethylformamide (DMF) solutions were prepared by the reaction of stoichiometric amounts of lead (II) bromide (PbBr_2_) and methylammonium bromide (CH_3_NH_3_Br). The dry powder forms of PbBr_2_ and CH_3_NH_3_Br were mixed before gradually adding to the DMF, while the solution was stirred and kept in room temperature to prevent clumping. In a typical synthesis, 1000.0 g of PbBr_2_ was reacted with 304.9 g MABr in 1400 mL of DMF. Single crystals were grown by inverse temperature crystallization from its saturated solution in DMF. The growth temperature was typically from 30 to 80 °C in the growing rate of 0.2–1 °C h^−1^. The temperature was controlled using a computer programmed and controlled magnetic stirrer hotplate and a stirred oil bath set up to achieve controlled heating.

##### Suspended Crystal Growth

A previously grown, seed single crystal of MAPbBr_3_, was attached to a cotton string and suspended in a saturated solution of MAPbBr_3_ as in the case of the inverse temperature crystallization method. Applying a temperature gradient to the solution and maintaining the concentration of the precursor (Figure S16, Supporting Information), this “suspended seed crystal” technique allows the seed to grow in all directions, easily reaching sizes of 3 cm. However, the single crystal will grow upwards too, starting to engulf the string itself. By varying the temperature very slowly (1 °C per hour), this can be controlled correctly to prevent any unwanted polycrystallization on the top of the single crystal, near the string. When removed from the solution the string can be taken out mechanically, with any new seed single crystals that appeared near the crystal surface.

##### OC2G

In the OC2G technique, good‐quality cubic shape SCs of MAPbBr_3_ was precisely aligned side‐by‐side along with their facets, just as a mosaic, or a 3D Rubik's cube pattern. It was not required to have SCs of the same size, however, attention was given on the perfect alignment of the edges to achieve an accurate and fast intergrowth. After immersing them into a solution of MAPbBr_3_ in DMF at 30 °C, they were fused together by inverse temperature crystallization. Under controlled and slowly increased temperature (1 °C every 2–3 h, Figure S17, Supporting Information), the solution gradually reached saturation and the growing crystals filled up the space between the aligned facets, resulting in a very firm connection without inducing any unwanted polycrystallinity. From 2 to up to 4 crystals were intergrowth by OC2G at the same time depending on the size and weight of the individual single crystals. Repeatable cycles, from room temperature to for example 80 °C (above dissolution can start), were done to achieve a better junction. Time of growth depended on the size and weight of the building blocks and lasted from a day to intergrowth 2–3 seed crystals of less than 20 g, to 2 weeks if merging two 200 g plates.

##### μ*τ* Product Measurements

The ***μτ*** product can be extrapolated from the voltage dependence of the photocurrent under gamma irradiation according to the Hecht equation^[^ ^11^, ^48^ ^]^

(1) $$\begin{array}{c} I=\frac{I_{0}\mu \tau V}{L^{2}}\frac{1-e^{\left(-\frac{L^{2}}{\mu \tau V}\right)}}{1+\frac{Ls}{V\mu }} \end{array}$$

where ***I***_0_ is the saturated photocurrent, ***L*** is the thickness, ***V*** is the applied voltage, and ***s*** represents the surface recombination velocity that directly affects the charge collection efficiency. For all the fabricated devices, the ***IV*** characteristics were acquired when exposed to a 2.3 Gy h^−1^ gamma dose‐rate from a ^60^Co source, in the applied voltage range of 0–100 V and fitted according to (Equation (1)).

##### Electronic Measurements

All measurements of the *I–V* characteristics and time evolution of the current of the device were done in ambient conditions at room temperature. The device characteristics have been determined by two‐point resistivity measurements, using golden wires contacted with either Dupont 4929 silver epoxy or carbon paste as electrical leads. A Keithley 2400 source meter allowed to measure the current with <0.1 nA resolution, while tuning the applied bias voltage, in dark and under gamma radiation.

##### Gamma Irradiation

All measurements were done inside an irradiation cavity “LOTUS” in the Laboratory for Reactor Physics and System Behavior at EPFL. It was equipped with a ^60^Co radiative source and a shielded irradiator that allowed safe usage of the source and irradiation with a horizontal collimated beam (15° opening angle). The ^60^Co source had an activity of 269 GBq and produced *γ*‐rays with energies of 1.173 and 1.332 MeV. The source was located under the cavity in the storage position and was brought into the cavity on a source rod to the exposed position in less than a second. In the exposed position, the source was held in front of the beam port. The gamma detector devices were positioned in the beam line at various distance from 25 to 125 cm from the source, thus exposing them to the corresponding dose rates from 0.07 to 2.3 Gy h^−1^. Long coaxial cables were used to connect the samples with the measuring setup outside the cavity.

## Conflict of Interest

The authors declare no conflict of interest.

## Supporting information

Supporting Information

Supplemental Video S1
