# Supplementary material for: Kilogram‐Scale Crystallogenesis of Halide Perovskites for Gamma‐Rays Dose Rate Measurements
Source: Adv Sci (Weinh). 2020 Dec 9;8(2):2001882. doi: 10.1002/advs.202001882 (PMC7816716; doi:10.1002/advs.202001882)
Supplement: Supplementary file 1 — Supporting Information [file ADVS-8-2001882-s001.pdf]

© 2020 Wiley-VCH GmbH

ADVANCED HEALTHCARE MATERIALS

Supporting Information

for *Adv. Healthcare Mater.*, DOI: 10.1002/advs.202001882

Kilogram-Scale Crystallogenesis of Halide Perovskites for Gamma-rays Dose Rate Measurements

Pavao Andričević, Pavel Frajtag, Vincent Pierre Lamirand, Andreas Pautz, Márton Kollár, Bálint Náfrádi, Andrzej Sienkiewicz, Tonko Garma, László Forró,* and Endre Horváth*

Supporting Information

Kilogram-Scale Crystallogenesis of Halide Perovskites for Gamma-rays Dose Rate Measurements

Pavao Andričević, Pavel Frajtag, Vincent Pierre Lamirand, Andreas Pautz, Márton Kollár, Bálint Náfrádi, Andrzej Sienkiewicz, Tonko Garma, László Forró*, Endre Horváth*

Dr. P. Andričević, Dr. M. Kollár, Dr. B. Náfrádi, Dr. A. Sienkiewicz, Prof. L. Forró, Dr. E. Horváth
Laboratory of Physics of Complex Matter (LPMC), Ecole Polytechnique Fédérale de Lausanne, Centre Est, Station 3, CH-1015 Lausanne, Switzerland
E-mail: pavao.andricevic@epfl.ch, laszlo.forro@epfl.ch
Dr. P. Frajtag, Dr. V. P. Lamirand, Prof. A. Pautz
Laboratory of Reactor Physics and Systems Behaviour, Ecole Polytechnique Fédérale de Lausanne, Centre Est, Station 3, CH-1015 Lausanne, Switzerland
Prof. T. Garma
Power Engineering Department, Faculty of Electrical Engineering, Mechanical Engineering and Naval Architecture, University of Split, Split, Croatia

Keywords: perovskite gamma detection, dosimetry, kilogram scale crystalogenesis, operational stability, self-healing

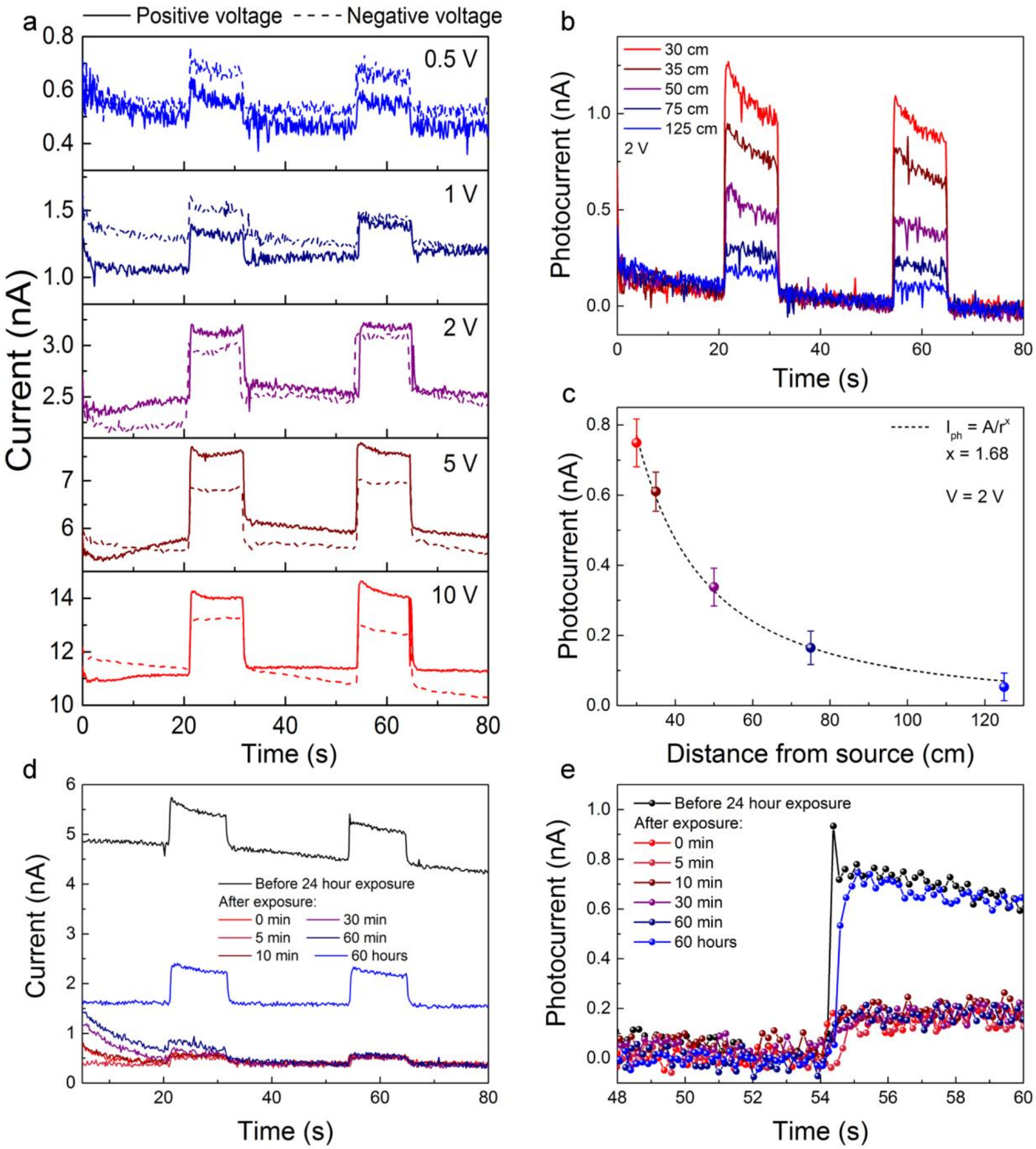

Figure S1. Performance of the Ag/MHP/Ag γ–ray photodetector. **(a)** Dark current and photocurrent stability under a 2.3 Gy/h dose-rate for different bias voltage. **(b)** On-off characteristics and **(c)** photocurrent dependence on the distances from the source fitted to the $\dot{D}_0/r^x$ function. **(d)** Comparison of current and **(e)** photocurrent response before and after 24

hours under operation and irradiation. Initial degradation of detection properties, as well as complete regeneration (after 60 hours) is visible.

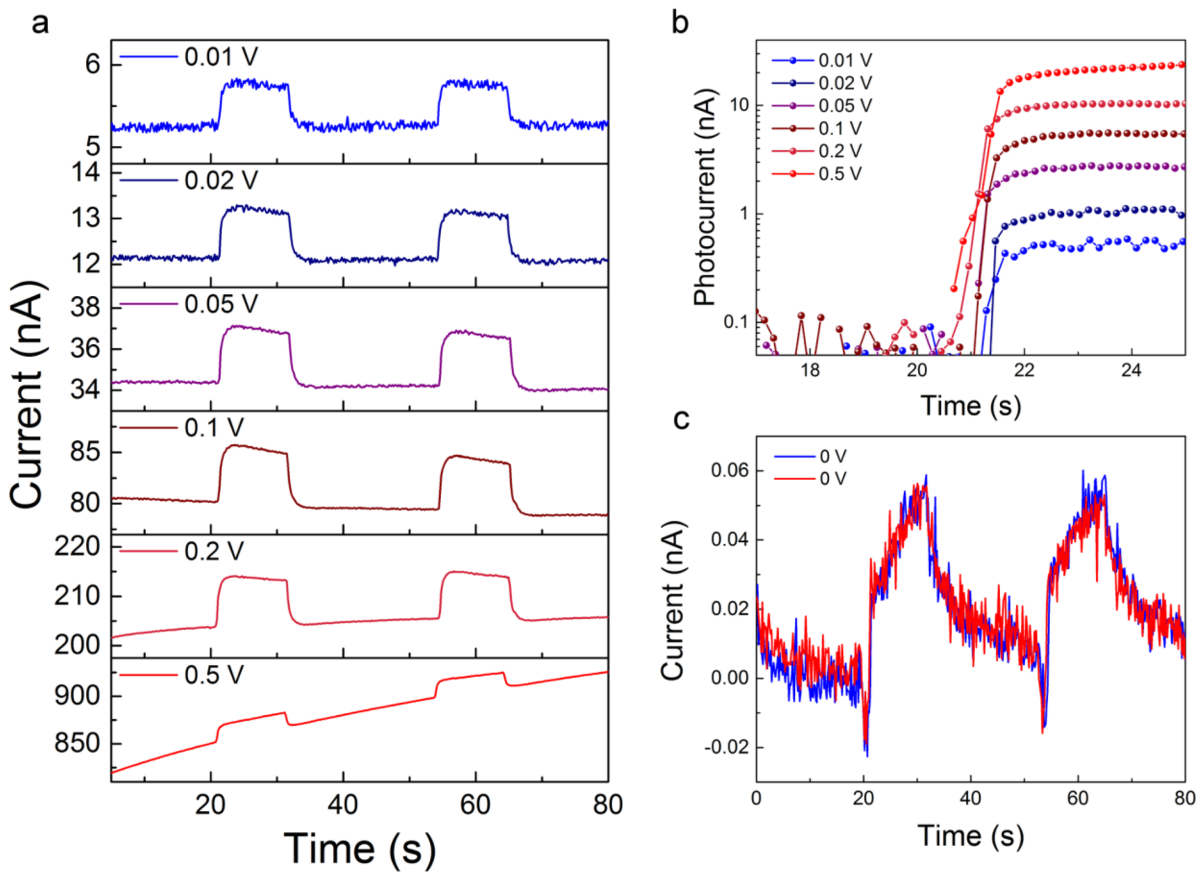

Figure S2. Performance of the VACNT/MHP/VACNT γ−ray photodetector. **(a)** Dark current and photocurrent stability under a 2.3 Gy/h dose-rate for different bias voltage. **(b)** Photocurrent response at different bias voltages as well as **(c)** 0 V, short circuit photocurrent.

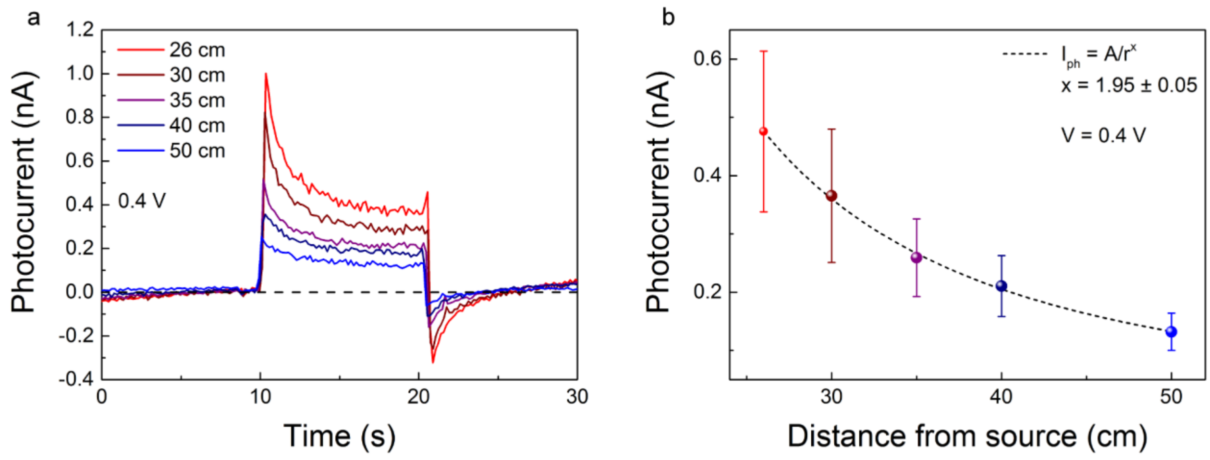

Figure S3 Dose-rate measurements of the VACNT/MHP/VACNT γ–ray photodetector. **(a)** On-off characteristics for different distances from the source. **(b)** Photocurrent dependence on the distance from the source fitted to the $\dot{D}_0/r^x$ function.

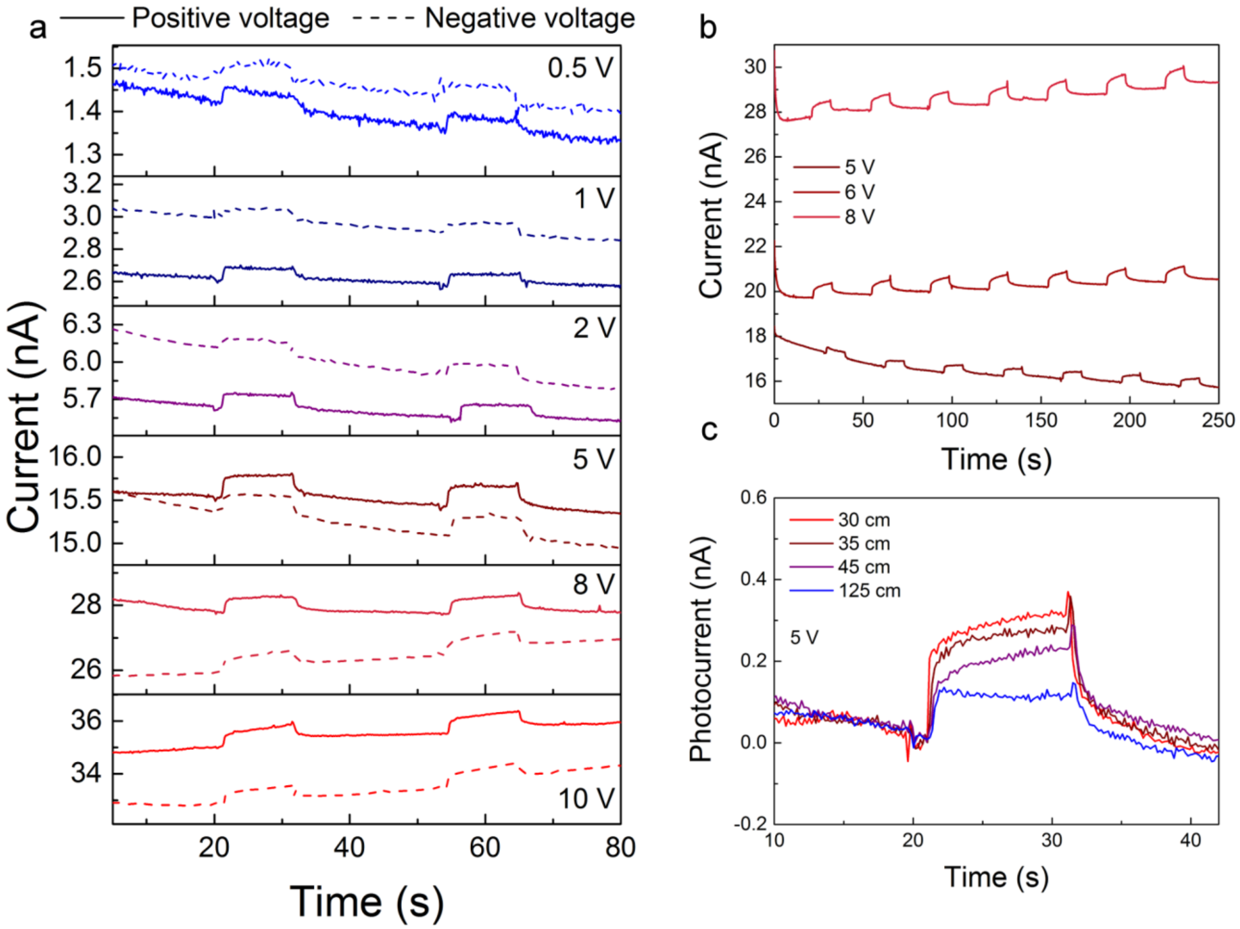

Figure S4. Performance of the carbon paper/MHP pallet γ−ray photodetector. **(a,b)** Dark current and photocurrent stability under a 2.3 Gy/h dose-rate for different bias voltage. **(c)** On-off characteristics for different distances from the source.

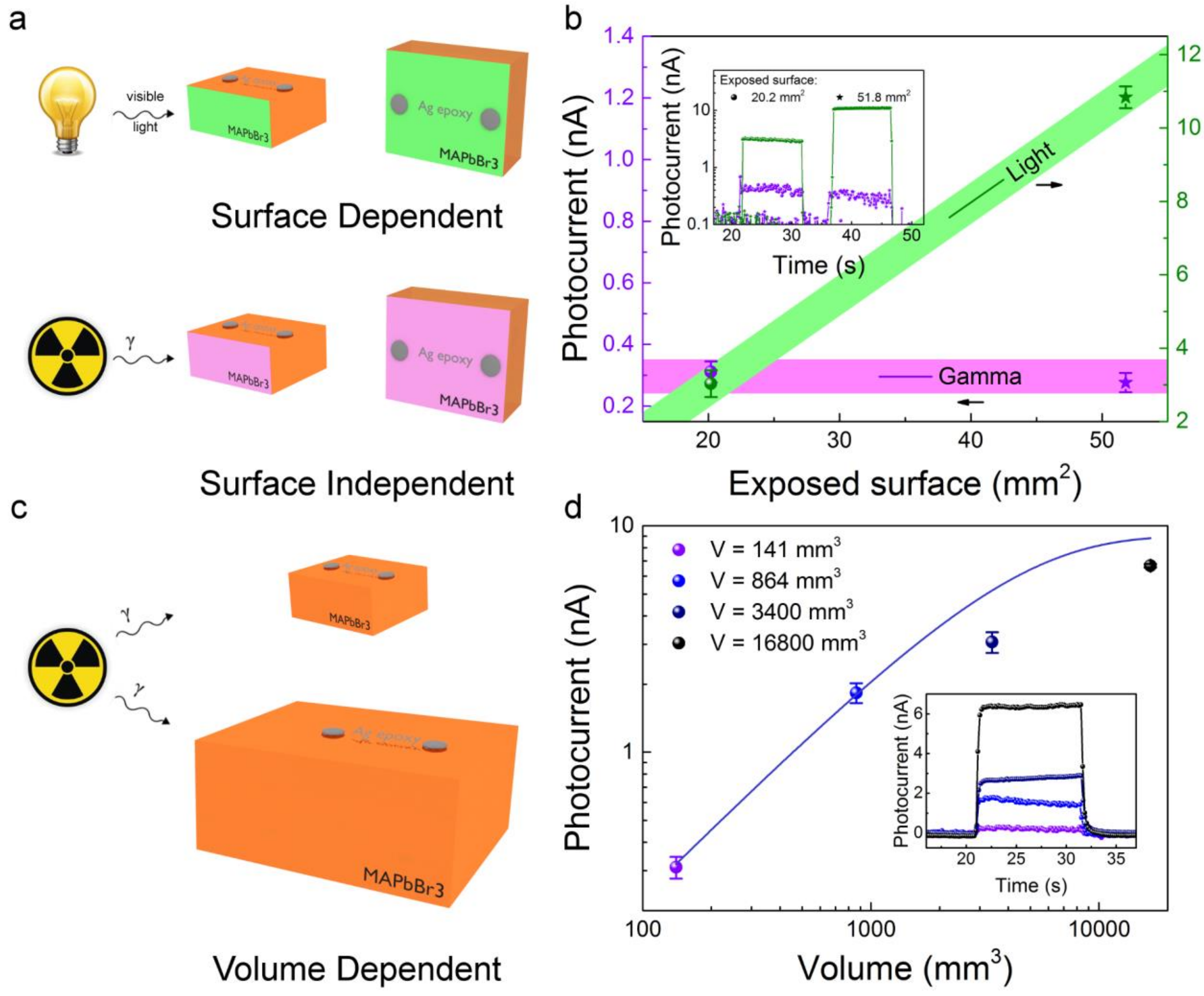

Figure S5. The size effect of perovskite based gamma detectors. (a) Schematic illustration of the MAPbBr3 SC-based detector exposed at different orientations to the incident *γ*-irradiation or visible light illumination. **(b)** Photocurrent dependence as a function of the exposed surface. Inset: On-off characteristics under gamma (purple) and white light (green). **(c)** Schematic illustration of the configuration of volume dependence measurements of the photocurrent **(d)** Photocurrent dependence as a function of the crystal volume. Blue line = guide for the eye. Inset: On-off characteristics.

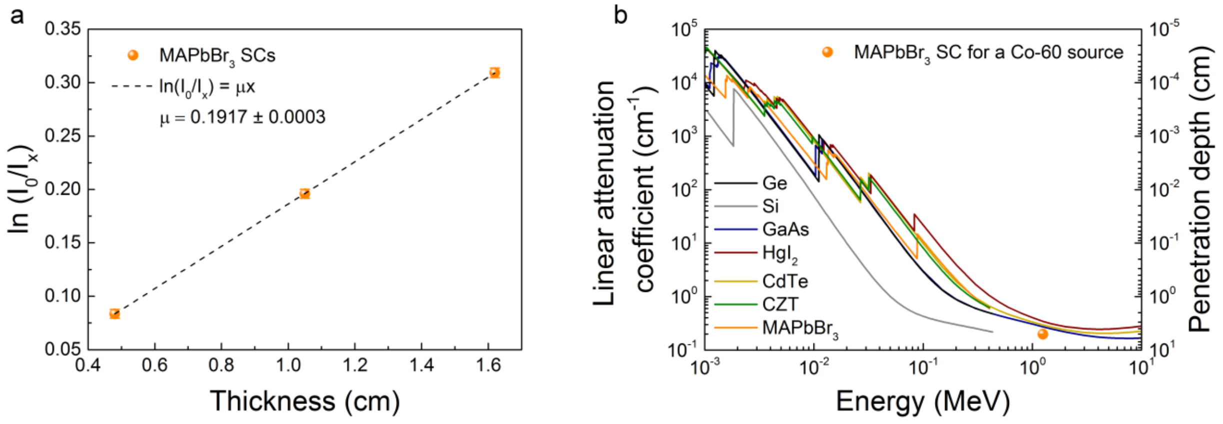

Figure S6. (a) Calculation of the linear attenuation coefficient of $MAPbBr_3$ SC for Cobalt 60 emitted gamma rays. (b) Linear attenuation coefficient and their corresponding penetration depth as a function of photon energy for different radiation detection materials from x to γ-ray.

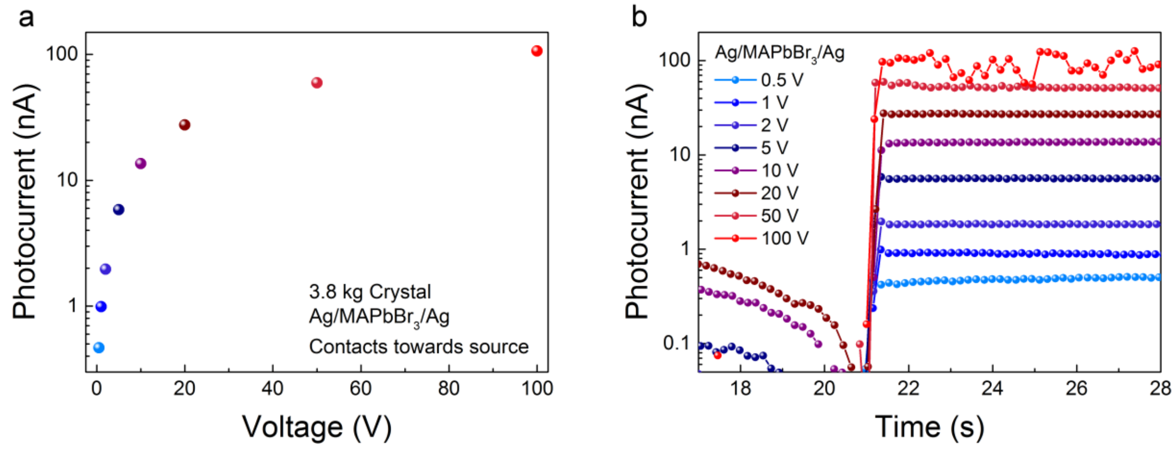

Figure S7. Performance of the Ag/OC2G crystal γ–ray photodetector. **(a,b)** Photocurrent dependence under a 1.25 Gy/h dose-rate for different bias voltage.

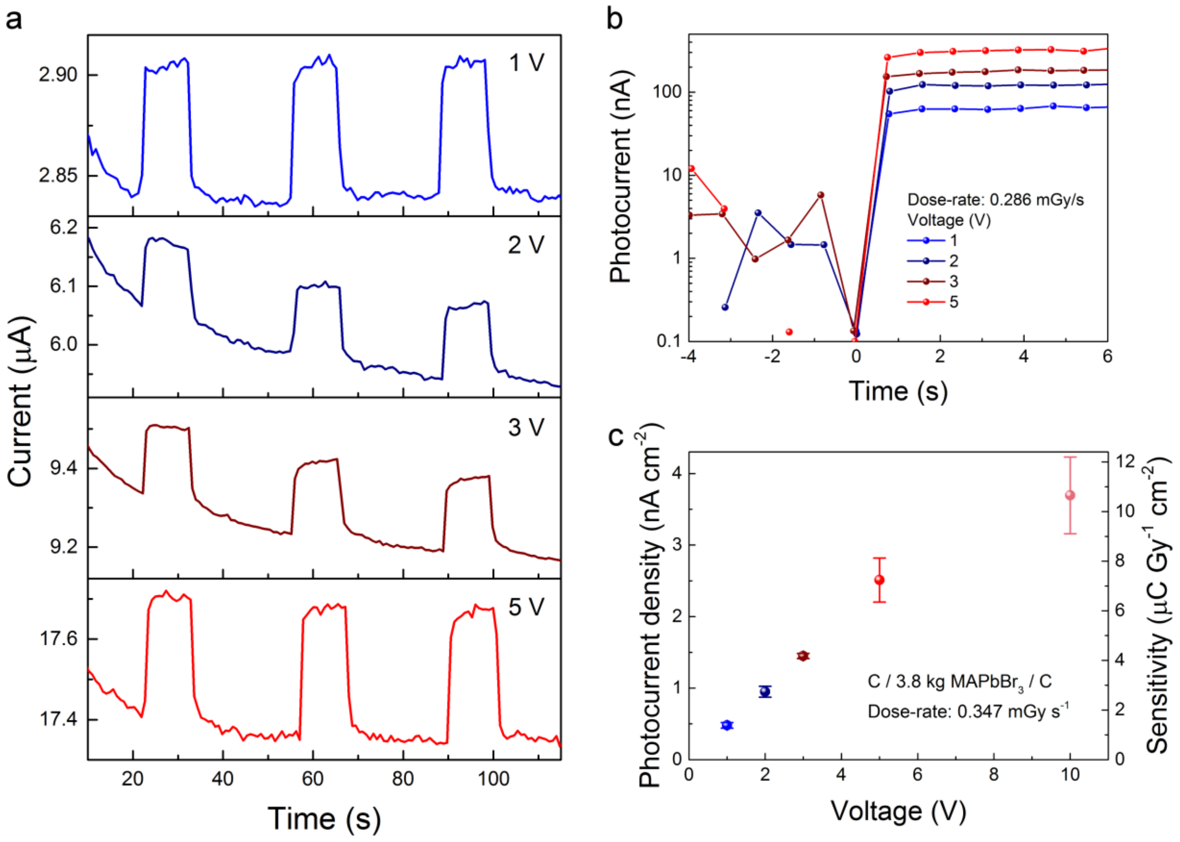

Figure S8. Performance of the Graphite spray/OC2G crystal γ−ray photodetector. **(a)** Dark current and photocurrent stability under a 1.25 Gy/h dose-rate for different bias voltage. **(b)** Photocurrent response and **(c)** sensitivity dependence with bias voltage.

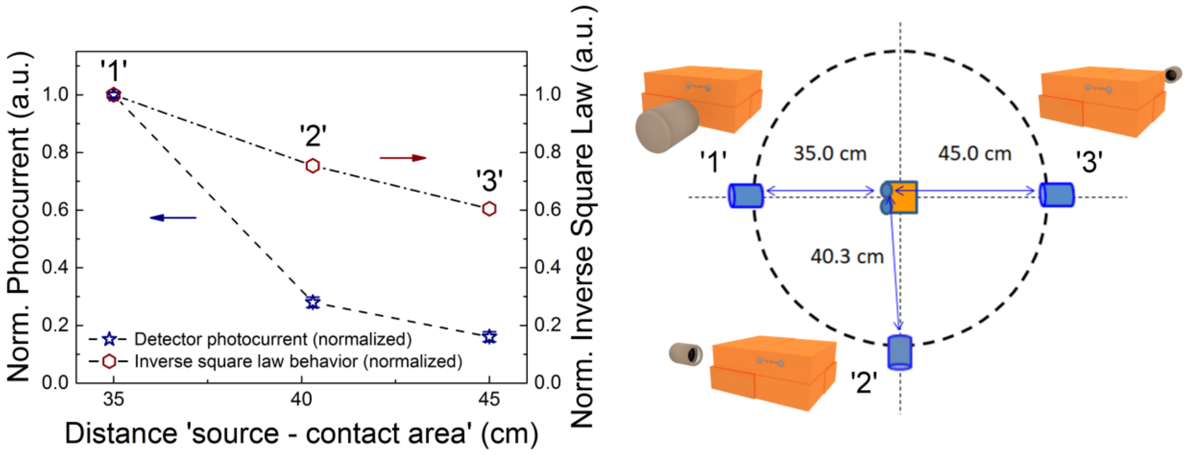

Figure S9. (a) Comparison of the evolution of the photocurrent across the gamma ray detector designed around a large $MAPbBr_3$ OC2G crystal (~1000 cm^3) and the 'inverse square law' corresponding to three different mutual orientations of the crystal facet containing the biasing electrodes and the radiation source (Cobalt-60). **(b)** Sketchy representation of the different mutual orientations of the radiation source and the gamma ray detector.

A thickness of 12 cm, can attenuate 93.5% of the 269 GBq ^{60}Co (125 MeV) source. To prove this assumption, we designed another detector based on the large OC2G crystal MAPbBr3 crystal (Volume ~1000 cm3). Two copper wires, embedded in silver-filled epoxy contact pads on one facet of the crystal, served as biasing electrodes. The detector was then positioned in the beam of gamma radiation in three different orientations. Firstly, the crystal facet containing the biasing electrodes was exposed directly to the source from a distance of 35 cm (orientation '1'). In this configuration, the 'active volume', within which the charges are collected most likely, was directly exposed. Next, the detector was rotated by 90°, thus moving the contact area further from the radiation source (orientation '2'). Lastly, the detector was turned by an additional 90°, thus positioning the contact area entirely opposite to the source (orientation '3'). In this latter arrangement, the crystal facet containing the biasing electrodes was at the distance of ~45 cm

from the radiation source, thus also being ‘shielded’ by the whole thickness of the $MAPbBr_3$ crystal (of ~ 10 cm).

the photocurrent across the detector drops markedly when the detector’s spatial position is being switched from the orientation ‘1’ to ‘2’. This pronounced drop of the photocurrent (of ~73%) can be associated with strong ‘shielding’ of the contact area provided by the outermost portion of the $MAPbBr_3$ crystal, which, for the orientation ‘2’, is directly facing the radiation source. The photocurrent continues to decrease (by dropping up to ~82%) also for the orientation ‘3’, in which, as mentioned above, the contact area of the detector is ‘shielded’ by the whole thickness of the MAPbBr3 crystal (~10 cm). It is worth noting that the herein reported drop of the photocurrent is definitely more pronounced than the one, which could be expected for an ‘inverse distance squared’ dependence (typically observed for exposure from a point radiation source). Altogether, these findings confirm strong attenuation of gamma rays by large-volume $MAPbBr_3$ crystals.

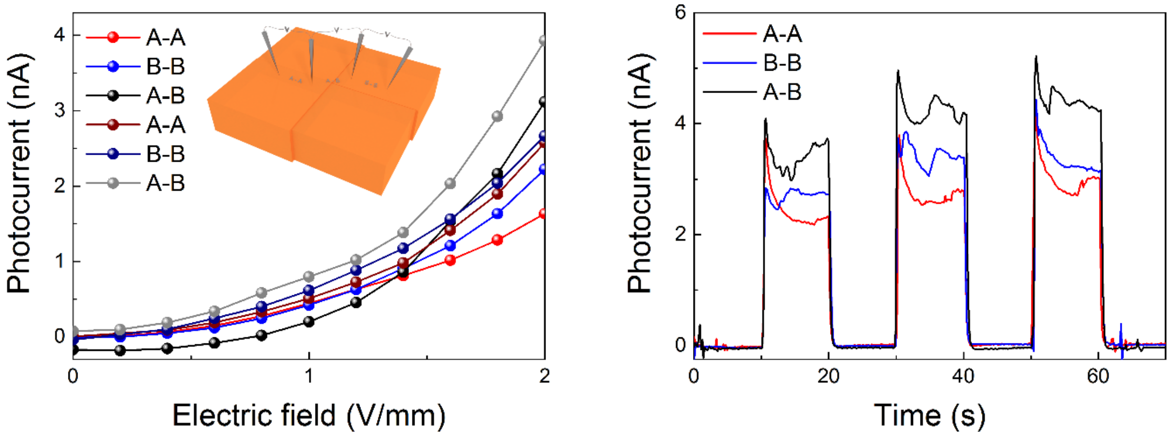

Figure S10. OC2G crystal boundary conduction. (a) Comparison of the current voltage characteristics, measured in the dark, inside a single block of the OC2G product (A-A, B-B) and through the boundary of the crystal blocks (A-B). (b) Photocurrent response under white light illumination for the different positions.

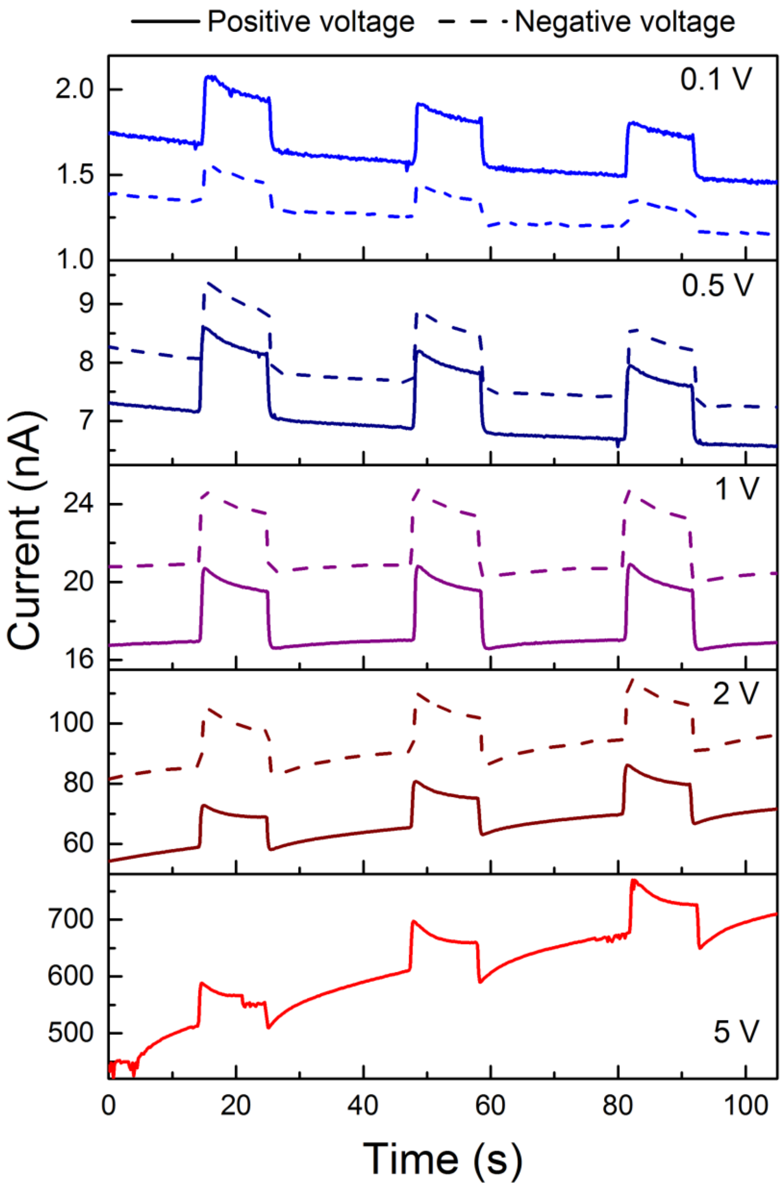

Figure S11. Performance of the Graphite spray/MHP SC γ–ray photodetector. Dark current and photocurrent stability under a 2.3 Gy/h dose-rate for different bias voltage.

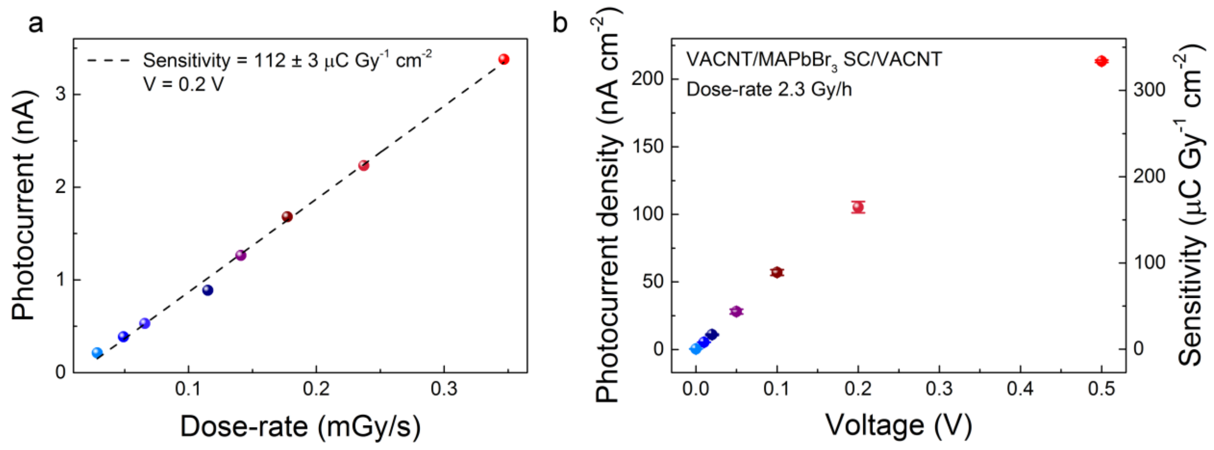

Figure S12. Sensitivity calculation for the VACNT/MHP SC γ–ray photodetector. **(a)** Estimated sensitivity from the linear fit of the photocurrent dose-rate dependence and **(b)** its change with bias voltage.

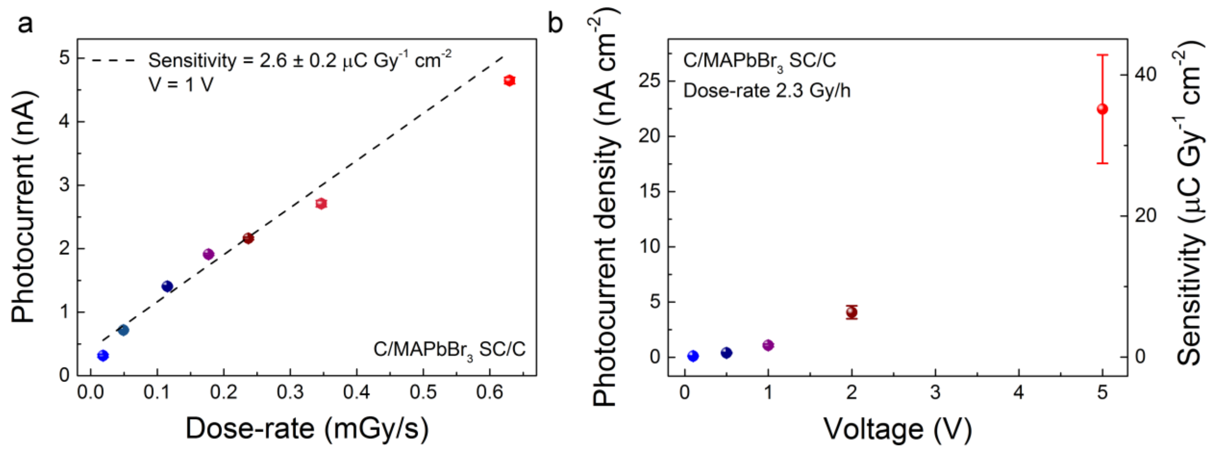

Figure S13. Sensitivity calculation for the Graphite spray/MHP SC γ–ray photodetector. **(a)** Estimated sensitivity from the linear fit of the photocurrent dose-rate dependence and **(b)** its change with bias voltage.

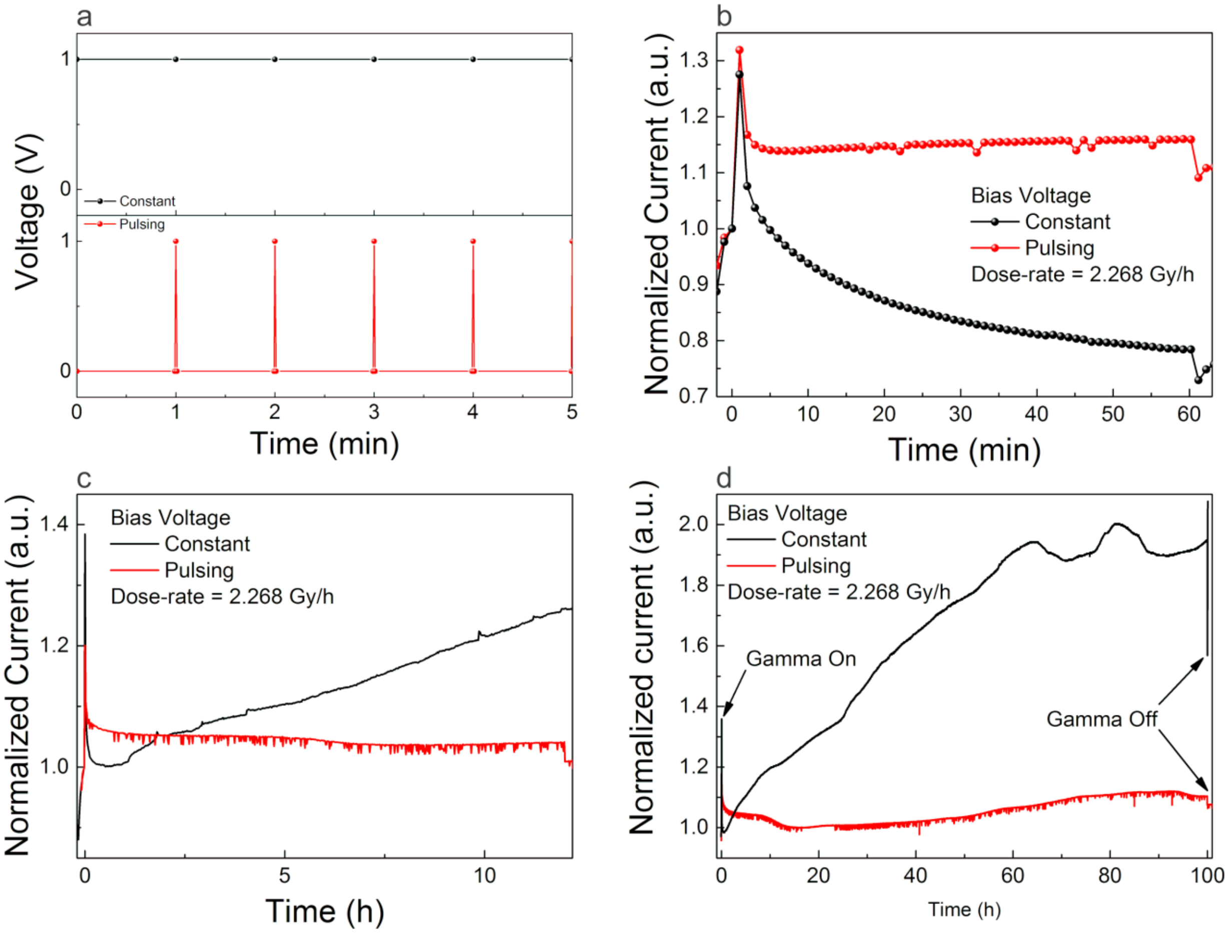

Figure S14. Long-term stability improvement of the Graphite spray/MHP SC γ–ray photodetector by pulsing the bias voltage. **(a)** Different bias voltage schemes. **(b)** Comparison of photocurrent stability in time for different bias voltage schemes under a 2.3 Gy/h dose-rate exposure for 1, **(c)** 12 and **(d)** 100 hours.

If the bias voltage is constantly applied in time, ion migration in the MHP crystal is strongly governing the drift of the current. However, when pulsing the voltage, the poling effect is suppressed and the device is stable in time under operational conditions.

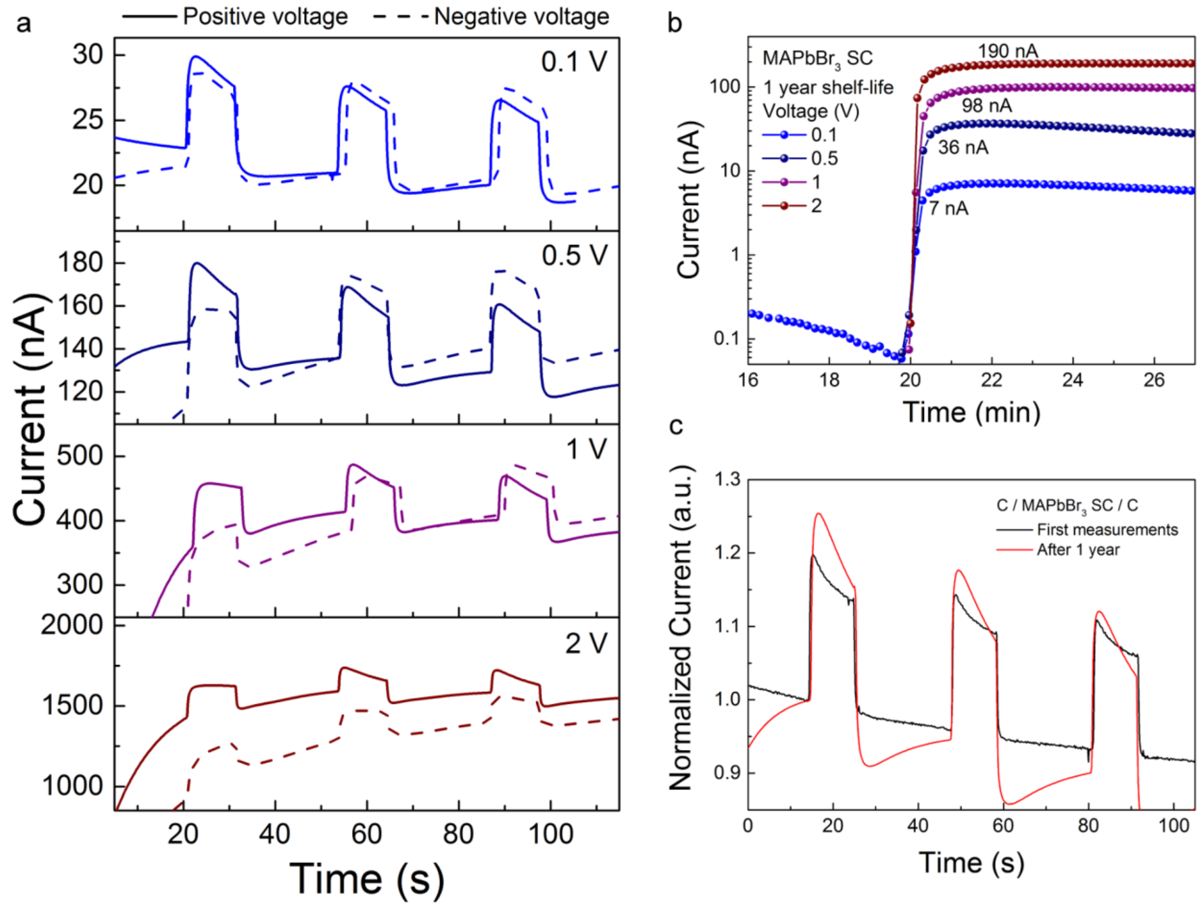

Figure S15. Performance of the Graphite spray/MHP SC γ–ray photodetector after 1 year. **(a)** Dark current and photocurrent stability under a 2.3 Gy/h dose-rate for different bias voltage. **(b)** Photocurrent response at different bias voltages. (c) Comparison to the first measurements obtained.

The measurements done after 1 year were performed after long-term testing which increased the dark current heavily due to ion migration in the crystal. This also increased the photocurrent of the device. Therefore, for a better comparison the current is normalized by the dark current.

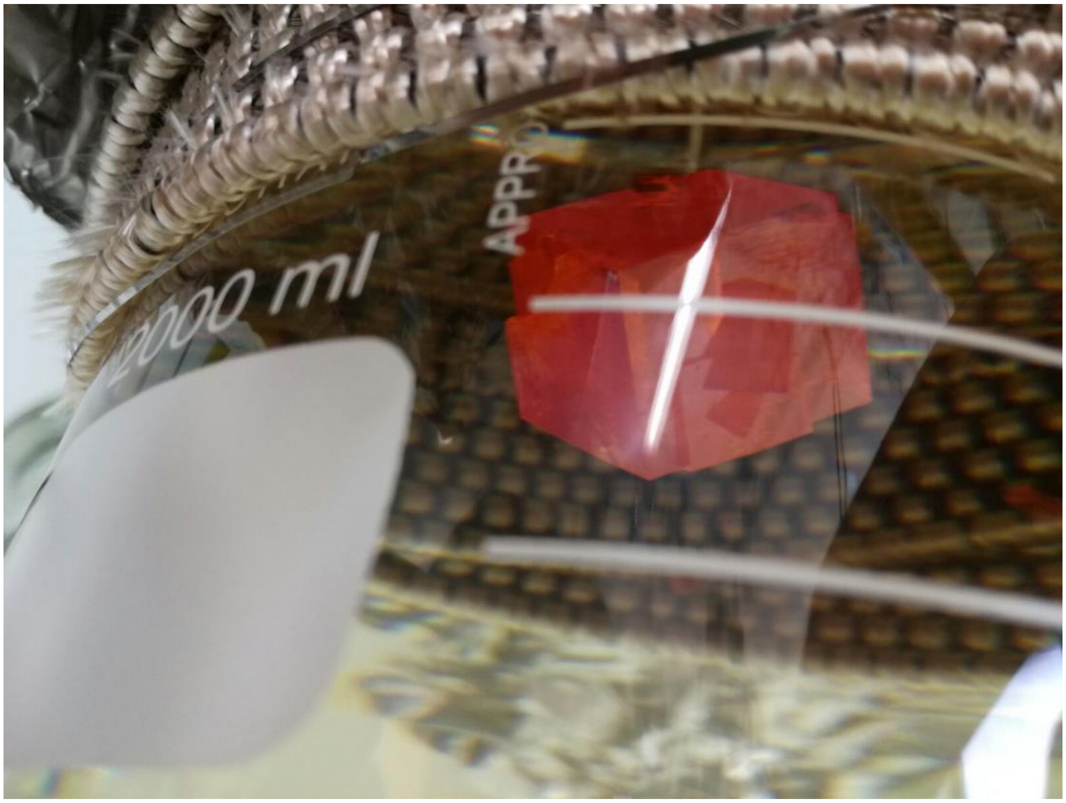

Figure S16. Optical image of the suspended crystal growth setup. A metal tube and heater are used to heat the beaker containing the solution from above and around.

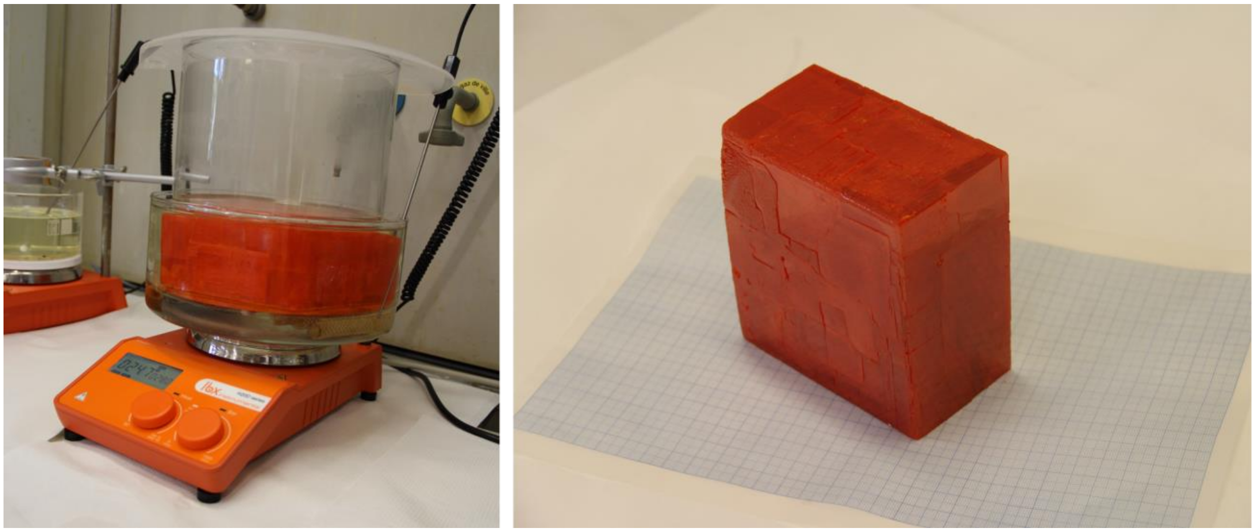

Figure S16. Optical image of the OC2 growth setup and the final crystal after smoothing of the edges.

Table S1. Comparative table of the performances of all perovskite-based gamma detectors.

Device structure	Sample size	μτ (cm^2/V) (Iph [illegible]	Source (Activity)	Energy (keV)	Dose rate (Gy/h)	Working Voltage (V)	Photocurrent (I_{ph}) Response [illegible]	Time to 90% [illegible]	Ref.
$CsPbBr_3$ single crystal	2.1 x 7 mm^2	1.3 x 10^{-3} (337 [illegible]	Ag (-)	21.69	-	450	-	-	[4]
Au / $MAPbI_3$ SC / PBCM:C60 /Ga Ga	3.3 - 10 mm	-	Cesium-137 (3774 GBq)	662	29	0	36.3 ± 0.3	-	[6]
$MAPbI_3$ SC	3 - 12 mm	1 - 1.8 x 10^{-3} (8keV X- [illegible]	Cesium-137 (2.2. MBq)	662	0.07	~ 100-1000	~ 0.005	~ 1	[7]
$MAPbI_3$ SC	3 - 12 mm	1 - 1.8 x 10^{-3} [illegible]	Carbon-11 (70 GBq)	960, 510	1	~ 100-1000	-	-	[7]
Cr / $MAPbBr_3$ SC / C60 / BCP / Cr	5 x 5 x 2 mm^3	0.4 - 1.6 x 10^{-3} (241Am)	Americium-241 (alpha, 29.6 GBq)	5480 (alpha)	1.1 x 10^{-4}	>180	-	-	[8]
$Cs_xFA_{1-x}PbI_{3-y}Br_y$ (x=0–0.1, y=0–0.6)	0.2 - 15 mm	4 x 10^{-2} - 1.2 x 10^{-1} (850 nm [illegible]	Americium-241 (0.4 MBq)	59.6	-		-	-	[9]
$Cs_xFA_{1-x}PbI_{3-y}Br_y$ (x=0–0.1, y=0–0.6)	0.2 - 15 mm	4 x 10^{-2} - 1.2 x 10^{-1} (850 nm [illegible]	Cesium-137 (2.2 MBq)	662	-		-	-	[9]
Cr / C60 / BCP /$MAPbBr_{2.94}Cl_{0.06}$ SC / Cr	14.4 x 13.7 x 5.8 mm^3	1.8 x 10^{-2} (390 nm light)	Cesium-137 (0.18 MBq)	662	1.4 x 10^{-6}	220	3000	< 0.5	[1]
Ga / $CsPbBr_3$/ Au	3 x 3 x 1 mm^3	1.34 × 10^{-3}	Cobalt-57 (185 MBq)	122	-	-150	-	-	[10] [12]
Ga / $CsPbBr_3$/ Au<br><br>Ga / $MAPbI_3$/ Au	4 x 2 x 1.2 mm^3<br><br>4 x 3 x 1.5 [illegible]	1.34 × 10^{-3} 8.1 × 10^{-4} (241Am)	Americium-241 (37 kBq)	59.6	-	-150<br><br>-50	-	-	[10] [12]
Ga / $CsPbBr_3$/ Au	4 x 2 x 1.24 mm^3	1.34 × 10^{-3} (241Am)	Cesium-137 (0.18 MBq)	662	-	-150	-	-	[10] [12]
Au / MAPbI3 / MAPbI3(Se - doped) / Au	10 x 10 x 4 mm^3	-	Cobalt-60	1250	108	0-200	~ 350	~ 5	[11]
C / $MAPbBr_3$ SC / C	34 x 37 x 43 mm^3	2.4 x 10^{-3} (60Co)	Cobalt-60 (269 GBq)	1250	0.07-2.3	0.1-20	2319.2	< 0.5	**This work**
C / $MAPbBr_3$ SC / C	120 x 120 x 70 mm^3	-	Cobalt-60 (269 GBq)	1250	1.25	1 - 10	309.05	< 0.5	**This work**

VACNT / $MAPbBr_3$ SC / VACNT	3 x 3 x 1 mm^3	0.5 x 10^{-3} (60Co)	Cobalt-60 (269 GBq)	1250	0.07-2.3	0.1-5	20	< 0.5	**This work**

Table S2. Comparative table of the performances of all gamma detectors tested in this work.

**Device structure**	**Sample size**	**Contact Distance (mm)**	**μτ (cm^2/V)**	**Dose rate (Gy/h)**	**Working Voltage (V)**	**Max Photocurrent Response (nA)**	**Max Photocurrent Density (nA cm^{-2})**	**Sensitivity (μC Gy^{-1} cm^{-2})**
Ag / $MAPbBr_3$ SC / Ag	12 x 12 x 6 mm^3	6	1.6 x 10^{-3}	0.07-2.3	0.5 - 10	1.25	-	-
VACNT / $MAPbBr_3$ SC / VACNT	3 x 3 x 1 mm^3	1	0.5 x 10^{-3}	0.07-2.3	0 - 0.5	20	213.3 ± 0.9	338.0 ± 0.4 (0.5 V)
C / $MAPbBr_3$ Pallet / C	28 x 6 mm^3	6	1.0 x 10^{-3}	0.07-2.3	0.5 - 10	0.3	~1	-
C / $MAPbBr_3$ SC / C	17 x 17 x 15 mm^3	3	2.4 x 10^{-3}	0.07-2.3	0.1 - 10	88.15	22 ± 5	2.6 ± 0.2 (1 V) 35 ± 8 (5 V)
C / $MAPbBr_3$ SC / C	34 x 37 x 43 mm^3	10	-	0.07-2.3	0.1 - 20	2319.2	184.3	49 ± 2 (2 V) ~530 (10 V)
Ag / $MAPbBr_3$ SC / Ag	120 x 120 x 70 mm^3	10	-	1.25	0.5 - 100	106.6	-	-
C / $MAPbBr_3$ SC / C	120 x 120 x 70 mm^3	10	-	1.25	1 - 10	309.05	3.69	10.64 (10 V)

Devices tested:

1) **Ag / $MAPbBr_3$ SC / Ag**

   12 x 12 x 6 mm^3

   6 mm contact distance

2) **VACNT / $MAPbBr_3$ SC / VACNT**

   3 x 3 x 1 mm^3

   1 mm contact distance

3) **Carbon paper / $MAPbBr_3$ Pallet / Carbon paper**

   28 x 6 mm^3

   6 mm contact distance

4) **Graphite spray / $MAPbBr_3$ SC / Graphite spray**

   17 x 17 x 15 mm^3

   3 mm contact distance

5) **Graphite spray / $MAPbBr_3$ SC 200g/ Graphite spray**

   34 x 37 x 43 mm^3

   10 mm contact distance

6) **Ag / OC2G $MAPbBr_3$ / Ag**

   120 x 120 x 70 mm^3

   10 mm contact distance

7) **Graphite spray / OC2G $MAPbBr_3$ / Graphite spray**

   120 x 120 x 70 mm^3

   10 mm contact distance

Degradation equation:[43]

$$\begin{aligned} CH_3NH_3PbBr_3 &\leftrightarrow [CH_3NH_3PbBr_{3+\delta}]^- + [CH_3NH_3PbBr_{3-\delta}]^+ \\ &\leftrightarrow PbBr_2 + \left[CH_3NH_3{}^+ + Br^-\right] \\ &\rightarrow\ PbBr_2 + CH_3NH_2 + HBr \end{aligned}$$

(1)
